# Carbon and Nitrogen Uptake of Calcareous Benthic Foraminifera along a Depth-Related Oxygen Gradient in the OMZ of the Arabian Sea

**DOI:** 10.3389/fmicb.2016.00071

**Published:** 2016-02-11

**Authors:** Annekatrin J. Enge, Julia Wukovits, Wolfgang Wanek, Margarete Watzka, Ursula F. M. Witte, William R. Hunter, Petra Heinz

**Affiliations:** ^1^Department of Palaeontology, University of ViennaVienna, Austria; ^2^Department of Microbiology and Ecosystem Science, University of ViennaVienna, Austria; ^3^Oceanlab, University of AberdeenAberdeen, UK; ^4^School of Biological Sciences, Queen's University BelfastBelfast, UK

**Keywords:** foraminifera, OMZ, Arabian Sea, carbon, nitrogen, *in situ*

## Abstract

Foraminifera are an important faunal element of the benthos in oxygen-depleted settings such as Oxygen Minimum Zones (OMZs) where they can play a relevant role in the processing of phytodetritus. We investigated the uptake of phytodetritus (labeled with ^13^C and ^15^N) by calcareous foraminifera in the 0–1 cm sediment horizon under different oxygen concentrations within the OMZ in the eastern Arabian Sea. The *in situ* tracer experiments were carried out along a depth transect on the Indian margin over a period of 4 to 10 days. The uptake of phytodetrital carbon within 4 days by all investigated species shows that phytodetritus is a relevant food source for foraminifera in OMZ sediments. The decrease of total carbon uptake from 540 to 1100 m suggests a higher demand for carbon by species in the low-oxygen core region of the OMZ or less food competition with macrofauna. Especially Uvigerinids showed high uptake of phytodetrital carbon at the lowest oxygenated site. Variation in the ratio of phytodetrital carbon to nitrogen between species and sites indicates that foraminiferal carbon and nitrogen use can be decoupled and different nutritional demands are found between species. Lower ratio of phytodetrital carbon and nitrogen at 540 m could hint for greater demand or storage of food-based nitrogen, ingestion, or hosting of bacteria under almost anoxic conditions. Shifts in the foraminiferal assemblage structure (controlled by oxygen or food availability) and in the presence of other benthic organisms are likely to account for observed changes in the processing of phytodetritus in the different OMZ habitats. Foraminifera dominate the short-term processing of phytodetritus in the OMZ core but are less important in the lower OMZ boundary region of the Indian margin as biological interactions and species distribution of foraminifera change with depth and oxygen levels.

## Introduction

Benthic foraminifera are unicellular protists found in marine sediments and occur with global distribution. The structure of benthic foraminiferal communities is controlled by environmental parameters, with oxygen and food (organic matter) availability being considered the most important factors (e.g., Jorissen et al., 1995). The impact of changing food flux and oxygen concentration on the abundance and distribution of species of foraminifera has been demonstrated in various studies from different regions (e.g., Heinz and Hemleben, 2006; Larkin and Gooday, 2009). The interplay between these complex environmental factors (organic matter, oxygen concentration) and the rank of influence of each factor on foraminiferal behavior often remain unclear. Investigations in oxygen-depleted ecosystems provide the opportunity to study foraminiferal faunas and their response to food under low oxygen (e.g., Woulds et al., 2007; Fontanier et al., 2014; Larkin et al., 2014).

Mid-water masses of minimum oxygen concentrations (<0.5 ml l^−1^ dissolved oxygen) intercept with the sea floor of continental margin at bathyal depths such as in the Arabian Sea (Diaz and Rosenberg, 1995; Levin, 2003) and expose sediments and organisms to severe and persistent oxygen depletion. Currently these Oxygen Minimum Zones (OMZs) affect 6% of the continental margin sea floor (Helly and Levin, 2004) but are predicted to expand due to human-induced eutrophication and global warming (e.g., Diaz and Rosenberg, 2008; Stramma et al., 2008). The largest OMZ is currently found in the Arabian Sea expanding from 120 to 1100 m water depth (Helly and Levin, 2004; Hunter et al., 2011) where foraminifera represent an important faunal component of the benthos and sustain dense populations (e.g., Levin, 2003; Gooday et al., 2009). Foraminiferal assemblages in oxygen-depleted environments are low in diversity and dominated by species with high stress tolerance and/or that benefit from adaptations such as increased pore density (e.g., Glock et al., 2011), denitrification (Risgaard-Petersen et al., 2006; Pina-Ochoa et al., 2010), and bacterial endobionts (e.g., Bernhard et al., 2012a,b; Nomaki et al., 2014). Observed elevated densities of foraminifera in OMZs have been also related to the absence of predators, and the high flux of organic matter to the sea floor at these depths (Levin, 2003).

Information remains scarce, however, on the role of foraminifera in the carbon cycling in the OMZ of the Arabian Sea with only few feeding experiments performed on the Indian margin (Moodley et al., 2011; Enge et al., 2014) and Pakistan Margin in the northern Arabian Sea (Woulds et al., 2007; Andersson et al., 2008; Larkin and Gooday, 2009; Larkin et al., 2014; Jeffreys et al., 2015). One approach of studying the feeding behavior under natural conditions are *in situ* feeding experiments where a food source (e.g., diatoms) is applied directly to the sea floor and left for ingestion by the specific target organism. After phytoplankton blooms, remains of microalgae (phytodetritus) can be found on the sea floor in a low degraded state. Such food source of high quality has been observed at 4000 m depth in the Arabian Sea (Pfannkuche et al., 2000). In the deep sea phytodetritus is a relevant food source for benthic foraminifera (e.g., Suhr et al., 2003; Nomaki et al., 2006) and its deposition can lead to shifts in distribution patterns and reproduction of foraminifera (Gooday, 1988, 1993). Phytodetritus “labeled” with stable isotopes (e.g., ^13^C) can be used in feeding experiments to directly track the food source to the consumer and to quantify uptake and uptake rates. The approach is very suitable for *in situ* feeding experiments and has been successfully performed in previous experiments to study the feeding behavior of deep-sea foraminifera (e.g., Levin et al., 1999; Moodley et al., 2002; Witte et al., 2003; Nomaki et al., 2006, 2011; Enge et al., 2011). In this study we used dual-labeled algae (^13^C and ^15^N) which allows us to simultaneously follow the uptake of carbon and nitrogen (Evrard et al., 2010). Nitrogen is often limited in marine environments and can restrict the growth of organisms as it is needed for the synthesis of amino acids and proteins.

The results presented here relate to the uptake by calcareous foraminifera from the 0–1 cm sediment horizon at 800 and 1100 m water depth. Calcareous species are the most important foraminiferal group in OMZ sediments and the majority of living foraminifera in OMZ sediments in the Arabian Sea is found in the upper sediment layer (e.g., Jannink et al., 1998; Maas, 2000; Schumacher et al., 2007). Data will be compared to results from a feeding experiment run at 540 m in the same geographic region (Enge et al., 2014).

## Materials and methods

### Study area

**Figure 1:**
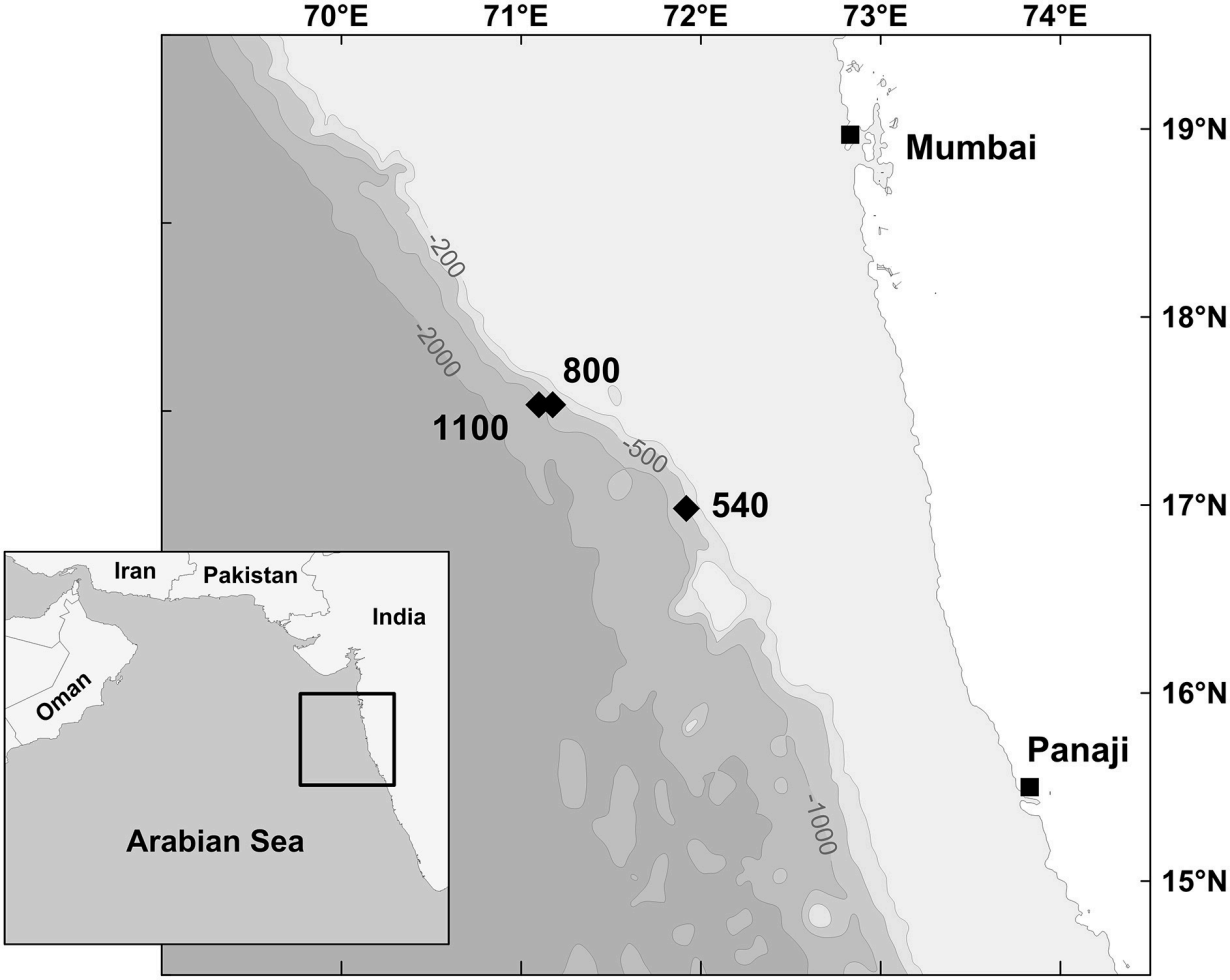
**Location of the experimental sites on the Indian Margin in the Arabian Sea**. Isobaths in meters.

This study is based on a set of *in situ* feeding experiments performed during the cruise “YK08-11” with the R/V *Yokosuka* (JAMSTEC, Japan). The experiments were conducted in an area located around 17°N and 71°E in the eastern Arabian Sea about 200 km off the coast of India (Figure 1). The area of interest shows an average productivity of ~0.5 g C m^−2^ d^−1^ with strong seasonal variation (Babu et al., 1999). The intense biological productivity in the Arabian Sea is driven by monsoon-induced upwelling in summer (SW monsoon) and by deeper mixing of water masses in winter (NE monsoon). The vertical flux of particulate organic carbon (POC) to the sea floor hence also shows seasonal variability. The annual POC flux in the eastern Arabian Sea estimates for 3.3 mg m^−2^ d^−1^ (Pfannkuche et al., 2000). The cruise took place between September and November 2008 during the post-monsoonal period. Environmental data for the experimental sites were obtained during the dives of the submersible *Shinkai 6500* (JAMSTEC, 2007), and derive from CTD recordings and measurements with an optical oxygen sensor. Calibrated oxygen concentrations, temperature, salinity, and sediment characteristics of each station are given in Table 1. The location of the experimental sites (540, 800, 1100 m) are within the range of the OMZ on the Indian margin (Hunter et al., 2011). The 540 m site is located in the OMZ core region showing the lowest bottom-water concentration of all investigated sites (<0.01 ml l^−1^ O_2_). The 800 and 1100 m sites are more oxygenated and are representative for the lower OMZ region of the Indian margin.

**Table 1:** **Environmental conditions at the experimental sites**.

	**Depth (m)**	**Duration (days)**	**Oxygen**		**Temperature (°C)**	**Salinity (PSU)**	**Sedimentary organic matter**		
			**(ml l^−1^)**	**(μmol l^−1^)**			**% POC**	**% TN**	**C:N**
540/4	552	4	<0.01	0.35	12.1	35.2	3.16	0.33	9.56
800/4	813	4	0.05	2.36	9.9	35.1	3.27	0.38	8.70
800/7	814	7					3.36	0.38	8.91
800/10	814	10					3.08	0.35	8.71
1100/4	1155	4	0.33	15.00	7.2	34.9	3.60	0.39	9.14
1100/10	1147	10					3.99	0.42	9.43

*Values for oxygen, temperature, and salinity derive from Hunter et al. (2012a). Values POC and TN derive from the same spreader as sediment samples for foraminiferal analysis (except site 540/4) and represent the 0–1 cm sediment horizon. For the 540/4 site, sediment parameters represent the mean of two samples from different spreaders of the same location*.

### Preparation of ^13^C and ^15^N-labeled algae

*Thalassiosira weissflogii* is a cosmopolitan marine diatom and belongs to the genus *Thalassiosira* which is part of the phytoplankton community in the Arabian Sea off the coast of India (Sawant and Madhupratap, 1996). Prior to the cruise, an axenic clone of *T. weissflogii* (CCMP, Bigelow Laboratories for Ocean Science, USA) was cultured in artificial seawater and *L*1 culture medium. The medium was enriched with 99%—^13^C-bicarbonate (NaH^13^CO_3_, Cambridge Isotope Laboratories, Inc., USA) and 50%—^15^N-sodium nitrate (Na^15^NO_3_, Cambridge Isotope Laboratories, Inc., USA). Algae were cultured at 16°C for 28 days (light: dark = 16:8; 35 PSU), harvested by centrifugation (500 G; 30 min), sonicated (2000 Hz; 5 min), and rinsed three times in ultrapure water to remove inorganic salts and dissolved organic carbon. Finally harvested algae were lyophilized (−60°C; −0.0001 mbar; 24 h). The produced phytodetritus contained 27.75 atom%^13^C and 33.70 atom%^15^N and displayed a C/N ratio (C:N) of 4.06 (Hunter et al., 2012a).

### Experimental setup

The same experimental work method was conducted at all depths (540, 800, 1100 m). The *in situ* feeding experiments were carried out using Oceanlab spreader systems. These are transparent polycarbonate tubes (25 cm inner diameter) with removable lids. A container inside the lid holds the suspension of *T. weissflogii* and can be opened by pushing the trigger with the arm of the submersible. For more detailed description of the spreaders see Hunter et al. (2012a).

At the beginning of each experiment, a spreader was deployed on undisturbed sea floor by the submersible. The algae suspension (650 mg C m^−2^, 160 mg N m^−2^) was then released from the spreader lid and left to settle on the sediment surface. The offered dose simulates an event of high organic matter influx. Several hours later the lid was removed in order to avoid artificial anoxia. The applied food amount and the open system guaranteed optimal simulation of an arriving pulse of fresh food to the sea floor under natural conditions. Incubation of the sediment area within the spreader with labeled phytodetritus lasted 4, 7, and 10 days. Not all periods of incubation were feasible at all three water depths. The following experiments on foraminifera were successful conducted and are part of this study: 4 days incubation (540, 800, 1100 m), 7 days incubation (800 m), 10 days incubation (800, 1100 m; Table 1).

After the incubation period, three sediment cores (70 mm inner diameter) were taken from inside each spreader of which one was available for foraminiferal analysis and the others were used for further analyses (see e.g., Hunter et al., 2012a,b). In total, six sediment cores (no replication) were available for foraminiferal analysis: one for each treatment, including the 540 m which has been already studied (Enge et al., 2014). On board of the vessel sediment in the retrieved cores was immediately cut horizontally in 1 cm thick slices and frozen −80°C. Transport and storage of samples until further processing took place at −25°C.

### Sample preparation

In the laboratory, samples were washed with artificial seawater over a 125 μm mesh and residues were frozen at −25°C until further processing. Foraminifera were wet-picked from the residue in a petri dish on a cooling plate. The identification of “living” and “dead” specimens and their separation was based on the color of the cytoplasm, and on the presence of cohesive cytoplasmatic filling of, at least the oldest, chambers (e.g., Moodley et al., 2002; Nomaki et al., 2005). We scanned the entire 0–1 cm sediment sample (38.5 cm^3^) for foraminifera and picked all “living” specimens. All “living” calcareous specimens (miliolids not included) were later analyzed for their isotopic composition for this study. To perform isotope analysis on foraminiferal cytoplasm, a minimum biomass (0.7 mg dry weight after decalcification) was required per sample. Some species were represented in insufficient numbers or were too small in size or volume. Pooling of species at lower taxonomic level was therefore necessary, especially for samples from 1100 m where abundances were lowest.

Before further processing of foraminifera for isotope analysis, material, and foraminifera were cleansed from adhering organic contaminants. For this, glassware was combusted at 450°C (5 h), picking tools and tin cups were cleaned in a mix of CH_2_Cl_2_ and CH_3_OH (1:1, *v*:*v*), and foraminifera were carefully brushed and washed twice in filtered artificial seawater and once in distilled water. Foraminifera were then transferred into tin cups. Excess water in the cups was removed before drying foraminifera at 50°C. Subsequently, 10 μl HCl (4%) were added to foraminifera for decalcification. Afterwards, samples were kept at 50°C for two days to allow complete drying.

### Sample analysis and data treatment

The contents of total organic carbon (TOC) and nitrogen (TN) as well as ^13^C/^12^C and ^15^N/^14^N ratios of foraminiferal cytoplasm were measured at the Stable Isotope Laboratory at the University of Vienna for Environmental Research (SILVER). An isotope ratio mass spectrometer (DeltaPLUS, Thermo Finnigan) with interface (ConFlo II, Thermo Finnigan) was used in combination with an elemental analyzer (EA 1110, CE Instruments). The minimum amount of carbon and nitrogen to be reliably determined is 5 μg C and 1 μg N for not labeled samples. At the double amount of carbon and nitrogen, relative standard deviations RSDs of 0.05% and 0.02% for atom%^13^C and atom%^15^N, and RSDs of 7.5%, 3.3%, and 4.3% for carbon content, nitrogen content and C:N ratio are achieved. The atomic composition of carbon and nitrogen isotopes of the sample (atom%X_sample_) were calculated against an international reference material (R_standard_): the Vienna Pee Dee Belemnite for carbon (R_VPDB_ = 0.0112372) and atmospheric nitrogen for nitrogen (R_atmN_ = 0.0036765):

(1) $$\text{atom}\%{\text{X}}_{\text{sample}}=\frac{100\;\times \left(\frac{\delta \text{X}}{1000}\;+\;1\right)\;\times \;{\text{R}}_{\text{standard}}}{1\;+\;(\left(\frac{\delta \text{X}}{1000}\;+\;1\right)\;\times \;{\text{R}}_{\text{standard}})}$$

with X being ^13^C or ^15^N, respectively. Excess of ^13^C and ^15^N in samples above background reflects uptake of ^13^C or ^15^N. Excess (*E*_sample_) is the isotopic label content and was calculated as the difference between the fraction of ^13^C (^15^N) in the background and the sample (after Middelburg et al., 2000):

(2) $$\begin{array}{r} E_{sample}=\left(\text{atom\%}{\text{X}}_{\text{sample}}-\text{atom\%}{\text{X}}_{\text{background}}\right)∕100 \end{array}$$

Background (natural) isotope signatures of foraminifera (δ^13^C = −20.5, δ^15^N = 10.7) derive from calcareous foraminifera in OMZ influenced sediments of the Pakistan and Oman margin (Jeffreys et al., 2015). Uptake of the isotopes ^13^C and ^15^N (*I*_iso_) was determined as the product of excess (*E*_sample_) and the total content of organic carbon (TOC) or nitrogen (TN) of the sample, respectively. Uptake of total phytodetrital carbon (pC, ^12^C + ^13^C) and nitrogen (pN, ^14^N + ^15^N) was determined by:

(3) $$\begin{array}{c} I_{\text{phyto}}=I_{\text{iso}}/(\text{atom}\%{\text{X}}_{\text{alga}} \end{array}/100)$$

with *I*_phyto_ as the content of pC (pN), and atom%X_alga_ as the fractional content of ^13^C (^15^N) in the labeled phytodetritus (Moodley et al., 2000; Hunter et al., 2012a).

The sum of pC (pN) of all samples analyzed from one site (e.g., 800/4) represents the total uptake of pC (pN) by all calcareous foraminifera >125μm (excluding miliolids) found at the particular site because all “living” calcareous foraminifera found were analyzed. Except for the site 540/4 where results demonstrate not the entire assemblage response due to failed analyses (see Enge et al., 2014).

## Results

### Foraminiferal assemblages

Total abundances of living foraminifera (not including tubular agglutinated) were highest at 540 m with 3914 ind. 10 cm^−3^ (Enge et al., 2014). Abundances were lower at 800 m (249 ± 40 ind. 10 cm^−3^) and lowest at 1100 m (99 ± 3 ind. 10 cm^−3^). Calcareous foraminifera were the main assemblage element at all three water depths. The numerical dominance of calcareous foraminifera decreased with increasing water depth from 99.5% at 540 m to 60% at 1100 m, while agglutinated foraminifera become more abundant with greater water depth (0.5% at 540 m to 34.5% at 1100 m). Miliolid and organic-walled foraminifera each represented less than 5% of the total foraminiferal abundance at all investigated depths. The species composition of calcareous foraminifera varied strongly between depths. At 540 m the assemblage was dominated by *Bolivina* aff. *Bolivina dilatata, Cassidulina* sp., and seven other species. Together these nine species represent 95% of all living specimens in the uppermost cm (Enge et al., 2014). At 800 m *Epistominella rugosa, Bulimina* cf. *gibba, Bulimina aculeata, B*. aff. *B. dilatata*, and the Uvigerinids are the most abundant taxa. The assemblages at 1100 m were dominated by *Globobulimina* sp. (1100/4) and *Bulimina mexicana* (1100/10). The following investigations and results (carbon and nitrogen isotopic composition and uptake) refer to calcareous foraminifera.

### Uptake of phytodetrital carbon

**Figure 2:**
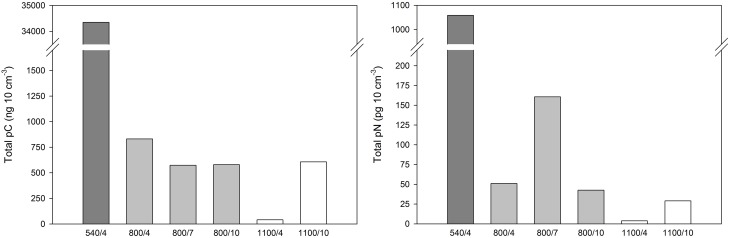
**Total content of phytodetritical carbon (pC) and nitrogen (pN) of calcareous foraminifera per 10 cm^**3**^ after 4–10 days of incubation with phytodetritus**.

**Figure 3:**
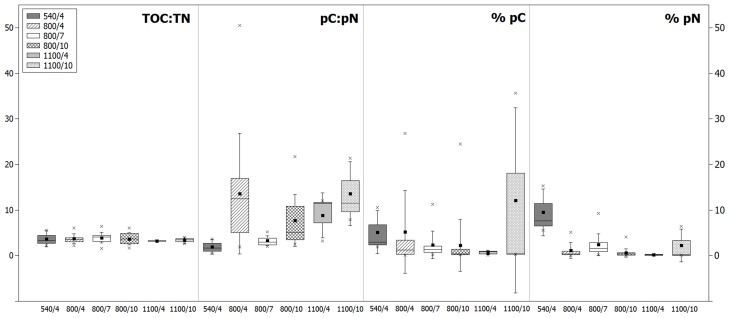
**The mean carbon and nitrogen stoichiometries of foraminifera for each treatment: the ratio of TOC and TN after feeding (TOC:TN), the ratio of pC and pN content in the cytoplasm (pC:pN), and the fraction of pC in TOC (% pC) and pN of TN (% pN)**. Box plots show the 25/75 percentile (*box*), mean (*filled square*), median (*straight line*), maximum and minimum (*crosses*), and the standard deviation (*whiskers*).

Total content of pC in foraminifera in the upper 1 cm sediment horizon after 4 days was 34.35 μg 10 cm^−3^ at 540 m, 0.83 μg cm^−3^ at 800 m, and 0.04 μg 10 cm^−3^ at 1100 m (Figure 2). The average fraction of pC (% pC) in foraminifera was highest at 1100 m after 10 days with great interspecific variation and lowest after 4 days at 1100 m (Figure 3).

**Figure 4:**
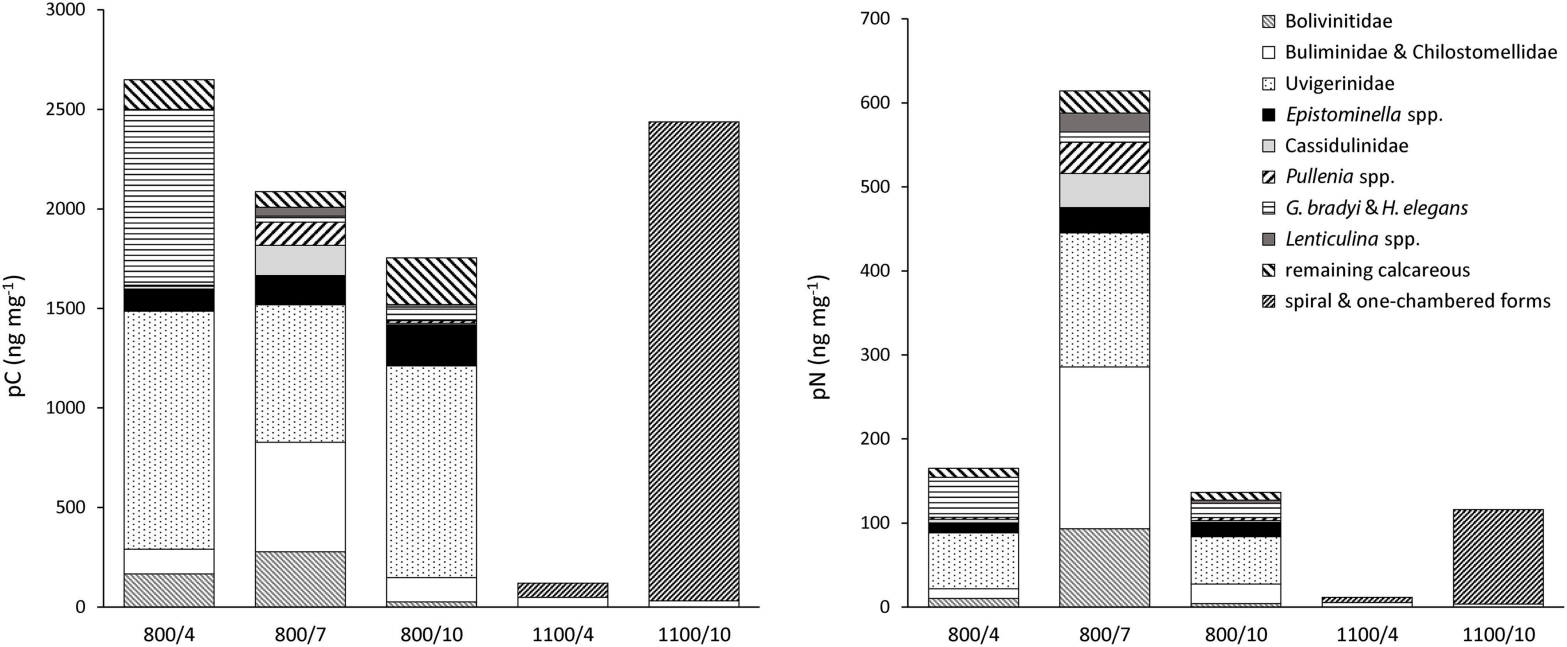
**Content of total pC and pN in foraminiferal species and groups at 800 m and 1100 m, normalized to sample weight (dry weight of analyzed foraminifera)**.

At 800 m, total pC was largest after 4 days and similar between 7 and 10 days (Figure 2). Content of pC varied largely between foraminiferal taxa (Table 2, Figure 4). Uvigerinids demonstrated the largest pC content of all groups at all three durations at 800 m (Figure 4). “*Gyroidina* spp., and *Hoeglundina elegans*” showed also high pC content after 4 days but with increasing time pC contents became lower (Figure 4). *Epistominella* species and the group of remaining calcareous foraminifera showed an increase in pC content over time while “Buliminidae and Chilostomellidae” showed largest pC content after 7 days (Figure 4). Highest fractions of pC at 800 m were present in “*Uvigerina schwageri* and *U. bifurcata*” (11.3–24.5%), in *B*. aff. *B. dilatata* (9.1% after 7 days), and in “*Gyroidina* spp. and *H. elegans*” (26.8% after 4 days).

**Table 2:** **Content of phytodetrital carbon (pC), nitrogen (pN), and the ratio of both in foraminifera species and groups after incubation with phytodetritus**.

	**4 days**			**7 days**			**10 days**		
	**pC (ng)**	**pN (ng)**	**pC:pN**	**pC (ng)**	**pN (ng)**	**pC:pN**	**pC (ng)**	**pN (ng)**	**pC:pN**
**540 m**									
*Bolivina dilatata*	1399.4	853.4	1.6	–	–	–	–	–	–
*Bolivina dilatata*	1499.0	–	1.8	–	–	–	–	–	–
*Bulimina gibba*	7966.5	2128.8	3.7	–	–	–	–	–	–
*Cassidulina* sp.	668.3	1091.3	0.6	–	–	–	–	–	–
*Cassidulina* sp.	481.5	–	0.4	–	–	–	–	–	–
*Ehrenbergina pacifica*	676.4	–	–	–	–	–	–	–	–
*Ehrenbergina pacifica*	364.2	–		–	–	–	–	–	–
*Epistominella rugosa*	1524.4	–	–	–	–	–	–	–	–
*Hoeglundina elegans*	10,804.9	–	–	–	–	–	–	–	–
*Lenticulina* sp.	1121.4	–	–	–	–	–	–	–	–
*Uvigerina schwageri*	93,088.0	–	–	–	–	–	–	–	–
*Uvigerina semiornata*	17,144.3	–	–	–	–	–	–	–	–
*Uvigerina semiornata*	13,112.8	–	–	–	–	–	–	–	–
**800 m**									
*Bolivina* aff. *B. dilatata*	–	–	–	202.2	66.8	3.0	–	–	–
(Other) Bolivinitidae	246.6	15.0	16.4	27.0	11.3	2.4	33.8	5.3	6.4
*Bulimina aculeata*	–	–	–	–	–	–	24.9	7.1	3.5
*Bulimina* cf. *gibba*	40.1	6.9	5.8	36.7	15.4	2.4	49.7	3.4	14.5
	40.3	0.8	52.0	152.4	56.6	2.7	22.1	3.0	7.3
	–	–	–	26.5	5.3	5.0	–	–	–
*Globobulimina* spp.	–	–	–	20.0	8.6	2.3	–	–	–
Other Buliminidae	75.8	6.7	11.4	62.2	15.8	3.9	14.6	3.8	3.8
Cassidulinidae	14.0	4.1	3.4	146.6	39.1	3.7	10.0	3.0	3.4
*Epistominella rugosa*	43.6	7.3	6.0	60.1	14.9	4.0	13.5	2.8	4.9
*Epistominella rugosa* and *E. exigua*	112.9	7.7	14.6	132.6	25.3	5.2	211.1	13.8	15.2
	–	–	–	–	–	–	54.5	4.7	11.7
*Gyroidina* spp. and *Hoeglundina elegans*	1064.6	56.8	18.7	41.8	16.8	2.5	–	–	–
*Gyroidina bradyi*	–	–	–	–	–	–	27.1	5.0	5.4
*Hoeglundina elegans*	–	–	–	–	–	–	9.1	3.6	2.5
*Pullenia* spp.	–	–	–	154.3	49.2	3.1	–	–	–
*Pullenia quinqueloba*	6.4	3.3	1.9	–	–	–	14.6	3.8	3.8
*Lenticulina* sp.	–	–	–	64.5	32.9	2.0	20.9	6.1	3.4
*Chilostomella* sp.	–	–	–	112.7	39.3	2.9	–	–	–
*Cancris* sp. and *Chilost*. sp. and *Globobul*. sp.	–	–	–	–	–	–	28.8	10.2	2.8
*Uvigerina schwageri* and *U. bifurcata*	1412.8	74.7	18.9	727.8	140.2	5.2	1405.6	64.7	21.7
*Uvigerina semiornata*	–	–	–	51.1	24.5	2.1	–	–	–
Other Uvigerinidae	7.6	3.7	2.1	108.3	29.2	3.7	17.8	4.2	4.3
	–	–	–	–	–	–	49.5	6.0	8.3
Remaining Calcareous	136.9	10.0	13.6	83.3	27.6	3.0	223.0	13.5	16.5
**1100 m**									
*Bulimina mexicana*	–	–	–	–	–	–	29.1	3.7	7.9
*Globobulimina* spp.	4.2	1.3	3.2	–	–	–	–	–	–
Other Buliminidae and *Chilostomella* sp.	72.6	6.1	12.0	–	–	–	18.8	1.6	11.5
Spiral shaped and one-chambered forms	80.5	7.1	11.4	–	–	–	2290.9	106.8	21.4

*Data from 540 m derive from Enge et al. (2014)*.

In contrast, at 1100 m the total content of pC in foraminifera had increased with time and it was almost 15 times the concentration after 10 days than after 4 days (Figure 2). Specific pC values of “Buliminidae and Chilostomellidae” slightly decreased between day 4 and 10 (Figure 4) while pC content of the “spiral shaped and one-chambered forms” group (including *G. bradyi, Pullenia* sp., *Epistominella* spp., *H. elegans*) had increased strongly from day 4 to day 10 (Figure 4). The “spiral shaped and one-chambered” group of 1100/10 demonstrated the highest fraction of pC of all investigated samples with 35.6%.

### Uptake of phytodetrital nitrogen

Total content of pN in foraminifera after 4 days was highest at 540 m (Figure 2) with only three species being measured and not the entire community such as at 800 and 1100 m. The average fraction of pN in foraminifera was also greatest at 540 m after 4 days, and lowest at 1100 m after 10 days (Figure 3). As observed for the carbon, contents of pN in foraminifera differed strongly between depths, incubation times, and species.

At 800 m, total pN in foramifera was similar between 4 and 10 days (0.04 and 0.05 μg 10 cm^−3^) and three to four times larger after 7 days (0.16 μg 10 cm^−3^; Figure 2). All species studied at 800 m showed their highest pN-values after 7 days except for “*G. brady*i and *H. elegans*” (Table 2). Uvigerinids dominated the total pN content at all three incubation periods (Figure 4). Highest fractions of pN at 800 m were observed in *B*. aff. *B. dilatata* (6.9%, 7 days), “*U. schwageri* and *Uvigerina bifurcate*” (9.3%, 7 days), and “*Gyroidina* spp. and *H. elegans*” (5.1%, 4 days).

At 1100 m, the total content of pN in foraminifera was 3.7 ng 10 cm^−3^ after 4 days and 29.1 ng 10 cm^−3^ after 10 days (Figure 2). After 10 days, “spiral shaped and one chambered forms” made up 95% of the total pN content at 1100 m (Figure 4) and also demonstrated highest fraction of pN at 1100 m (6.4%).

### The relationship between carbon and nitrogen uptake

**Figure 5:**
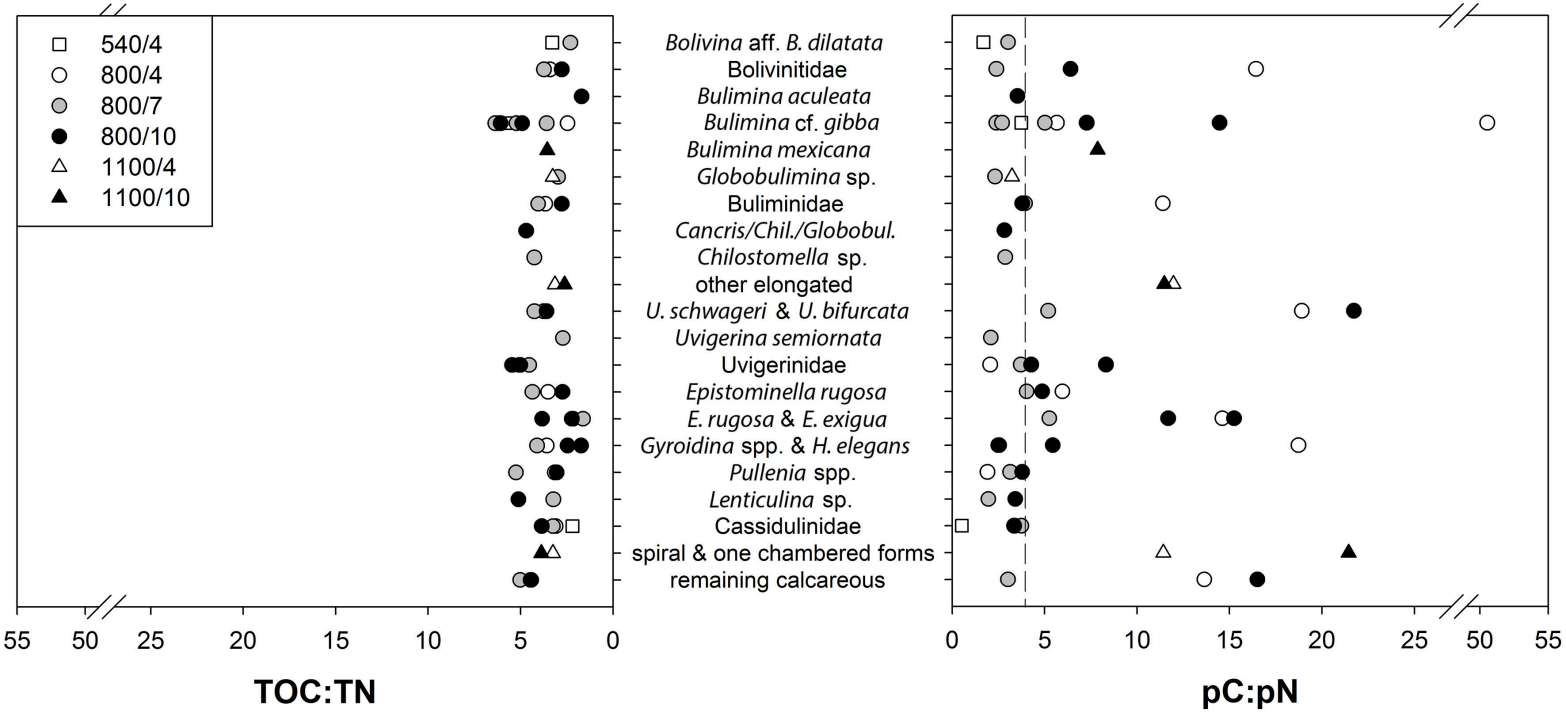
**Specific ratios of total and phytodetrital carbon and nitrogen content of foraminiferal species and groups after incubation with phytodetritus**. Dashed line represents C:N of the phytodetritus.

To study the relationship between uptake of carbon and nitrogen from phytodetritus, the ratio of total organic carbon to nitrogen (expressed as TOC:TN) and the ratio of pC and pN (expressed as pC:pN) were estimated. TOC and TN were measured on the same foraminifera as the isotopes. Hence, TOC and TN also contain phytodetrital carbon and nitrogen. But as the fraction of pC and pN is on average below 10% of TOC and TN (Figure 3), TOC:TN mainly reflects the carbon and nitrogen levels of the cytoplasm of foraminifera. Variation in TOC:TN ratios was low between species of one depth (Figure 5) and from different water depths (Figure 4). Foraminifera showed a mean TOC:TN of 3.7 (±1.2) over all three water depths. The mean TOC:TN ratio was similar at 540 and 800 m (3.7 and 3.8), but lower at 1100 m (3.3). The TOC:TN of all studied samples ranged between 1.6 for “*E. rugosa* and *Epistominella exigua*” and 6.4 for *B*. cf. *gibba*, both peak values observed in the 7 day treatment at 800 m.

The pC:pN showed greater variation between species and within sites than TOC:TN (Table 2, Figure 5). The pC:pN of foraminiferal taxa ranged between 0.6 for *Cassidulina* sp., (at 540/4) and 56.1 for *B*. cf. *gibba* (at 800/4). The mean pC:pN was lowest at 540/4 (2.4) and at 800/7 (3.9). At both sites, pC:pN was lower than TOC:TN. The mean pC:pN of foraminifera from the other treatments (800/4, 800/10, 1100/4, 1100/10) were larger than 9 (with a maximum of 16.3; Figure 3).

## Discussion

### Limitations and challenges

We performed *in situ* feeding experiments because they allow the observation of organisms and their reaction to induced changes under natural conditions. In contrast, observations on organisms gained during laboratory experiments can be biased by the artificial setting which differs from nature (e.g., pressure) and might not reflect the natural response (e.g., Woulds et al., 2007; Sweetman et al., 2009). But *in situ* experiments come with restrictions and problems, especially when performed at greater water depths. Restrictions in deep-sea research include limited diving time, limited space in transport box of the submersible, and therefore the limited number of samples. Problems like tipped spreaders during the release or recovery phase or a broken trigger can often not be fixed due to time limitation. Also measurements of duplicates failed which further reduced the already low replication to, in our case, one replicate per treatment (water depth-duration combination). We are aware that one sample cannot reflect the entire spectrum of possible responses by the foraminiferal assemblage and that trends need to be considered carefully. Still, our results provide insight in the ecology of foraminifera in an extreme marine ecosystem that is difficult to access and that has not been investigated from this point of view before.

Another challenge was the identification of “alive” and “dead” specimens. “Living” specimens were counted and picked out from the sediment for analysis of isotopic composition of the cytoplasm. Tearing of foraminiferal cytoplasm can be caused by fast transfer between very different pressure conditions (e.g., when recovering sediment from 1100 m to the water surface). The distinction between pressure-caused rupture of cytoplasm in “living” foraminifera and decay-related break up of cytoplasm in “dead” foraminifera was difficult. It is therefore possible that “living” specimens with ruptured cytoplasm were sorted as “dead.” Our counts might underestimate the actual abundance of living foraminifera on the Indian margin. Still total abundances are in the range of estimated foraminiferal densities in the OMZ off Pakistan and Oman (Gooday et al., 2000, 2009; Maas, 2000) and also the shift in assemblage composition with depth is similar with observations in other OMZs (Bernhard, 1992; Maas, 2000; Cowie and Levin, 2009; Caulle et al., 2014).

### Species specific food uptake

The high pC-values of *Uvigerina schwageri*, “*U. schwageri* and *U. bifurcate*” and “*Gyroidina* spp., and *H. elegans*” (Table 2) indicate strong uptake of *T. weissflogii* phytodetritus. High and rapid uptake of phytodetritus by *Uvigerina* species has been reported from OMZ sediments at the Pakistan Margin (Woulds et al., 2007; Larkin and Gooday, 2009; Larkin et al., 2014) and from the Sagami Bay (Nomaki et al., 2006). The dominance of the assemblage uptake demonstrates the important role of *U. schwageri* in processing of fresh phytodetritus. The strong response might result from refilling its deposits by ingesting as much food as possible as soon as it becomes available which would be very advantageous against food competitors. After 4 days feeding on *T. weissflogii*, 35% of the total carbon of *U. schwageri* derived from phytodetritus (Enge et al., 2014). Ingestion of phytodetritus of up to 40% its biomass within 9 days has been observed for *Uvigerina atikaensis* (Nomaki et al., 2011), also a species living under dysoxic conditions (Fontanier et al., 2014). Ingestion of *T. weissflogii* during the experiments created a beige/light brown coloration of the cytoplasm of the youngest chambers in several specimens. The greenish coloration of some individuals of *U. schwageri* and *H. elegans* at 540 and 800 m indicates the ingestion of a different phytodetritus source before or during the experiments and underlines the relevance of fresh phytodetritus in the diet of these species.

### Response to food over time

The content of pC in foraminifera at 800 m was highest after 4 days, and lower after 7 and 10 days (Figure 2). Part of phytodetrital carbon might have been already respired during the incubation period of 10 days, leading to lower pC contents despite carbon uptake. Lower content of pC with time could also hint for food saturation in some species after feeding for several days (e.g., Cassidulinidae, Bolivinidae). On the contrary, Uvigerinids showed similar high pC contents at all three incubation periods (Figure 4) which points again to continuous uptake of food by some species. The observed high pC levels in Uvigerinids after 10 days might have additionally resulted from migrating specimens from sediments below 1 cm toward the deposited phytodetritus near the sediment surface, indicated by slightly higher abundances of some uvigerinid species at 800/10 than at 800/4. Uvigerinids are infaunal species and also occur deeper than 1 cm of OMZ sediments (e.g., Maas, 2000; Schumacher et al., 2007; Caulle et al., 2014). Difference in abundance, however, caused by the patchy distribution of foraminifera in deep-sea sediments and given the missing replication, cannot be excluded.

At 1100 m, content of pC in foraminifera was higher after 10 days than after 4 days, suggesting an increase in uptake of phytodetritus over time. The retarded response to food, which has been observed for other deep-sea foraminifera (Witte et al., 2003), could result from a change of metabolic activity. Foraminifera at 1100 m might maintain a lower metabolic activitiy and energy level than at 800 m as adaptation to lower influx of particulate organic matter. The presence of organic matter can alter metabolic activity of certain deep-sea foraminifera to take quick advantage of sudden food availability (Linke, 1992).

### Role of foraminifera in phytodetritus processing within OMZ

The ingestion of pC by calcareous foraminifera was evident after 4 days at 540, 800, and 1100 m, but total carbon uptake varied greatly with depth with a clear maximum at 540 m. Differences in carbon uptake as result of food limitation can be excluded because the same amount of phytodetritus was applied at each site and on average only 2% of the total added carbon was found in form of pC in foraminifera after 4–10 days of feeding. The variation in total carbon uptake of foraminifera between water depths can result from different foraminiferal assemblages and biological interactions across the study area, caused by depth-related changes of environmental conditions (e.g., oxygen concentration).

At 540 m, foraminifera and bacteria are the only benthic organisms found alive on the Indian margin (Hunter et al., 2011, 2012a,b; Enge et al., 2014), representing the sole potential consumers of phytodetritus. The lack of macrofauna at 540 m represents a release from predation on foraminifera potentially causing higher abundances and biomass of foraminifera (e.g., Maas, 2000; Levin, 2003). Missing macrofauna additionally causes less competition for food which might have cause the strong response to *T. weissflogii* by foraminifera. Also, the assemblage of living foraminifera at the 540 m site is dominated by few species with representatives of the genera *Bolivina, Bulimina*, and *Uvigerina* (Enge et al., 2014). These taxa are typically found in OMZs (e.g., Maas, 2000; Schumacher et al., 2007) because they are stress tolerant and depend on high quantities of organic matter. The high demand for food, reflected by their dominance of total carbon uptake (89%) at 540 m, could be caused by higher metabolism rates to cope with the oxygen stress.

The flux of organic matter to the sea floor in the lower OMZ boundary region is lower than in the core region (Calvert et al., 1995). The different composition of the foraminifera assemblage might cause the decreased carbon uptake with depth. A third the foraminiferal assemblage at 1100 m is made up by agglutinated foraminifera who are often associated with more oligotrophic conditions and do not depend on high amounts of food to survive. Species that dominated carbon uptake at 540 and 800 m (*U. schwageri, B*. aff. *B. dilatata, G. bradyi*) were not present in the upper 1 cm sediment at 1100 m but might be found below the sediment surface where they could contribute to carbon uptake below 1 cm. Representatives of *Bolivina* and *Uvigerina* are found deeper distributed in the sediment at the boundaries of OMZs with better oxygenation (e.g., Schumacher et al., 2007). The lower total uptake at 1100 m by foraminifera might not only resulted from changed species composition and distribution, but also from increased predation and food competition by the macrofauna. The population density and uptake of phytodetritus of the macrofauna increases with greater water depth and better oxygenation from 540 to 1100 m on the Indian margin (Hunter et al., 2011, 2012a). The influence of the macrofauna upon bacterial activity across the Indian margin has been shown by Hunter et al. (2012b) and might also drive to some extent the cycling of organic matter by foraminifera.

The observed shift of dominance in the processing of phytodetritus from macrofauna to foraminifera with increasing oxygen depletion was also noted on the Pakistan margin (Woulds et al., 2007), and underlines the importance of foraminifera to carbon cycling under severe oxygen depletion.

### Uptake of nitrogen by foraminifera

The TOC:TN-values showed low variation between species and water depths (Figure 3), indicating a similar trophic level of foraminifera at the time of post-monsoonal sampling within the OMZ. Our observations resemble the C:N of benthic foraminifera from Sagami Bay at 1453 m water depth, ranging between 2.4 and 6.4, depending on sampling period before or after phytodetritus deposition in spring (Nomaki et al., 2006, 2011). Both sampling sites are located at eutrophic bathyal settings with sufficient food supply and available carbon and nitrogen for benthic foraminifera. Limitation of nitrogen would be reflected in a greater C:N.

The ratio of phytodetrital carbon to nitrogen content varied greatly (from 0.4 to 52.0) between species and groups (Table 2, Figure 5), reflecting different demands of carbon and nitrogen. Lowest mean pC:pN were shown at 540/4 and 800/7, which are not result of one or few dominant species but rather an assemblage trend, evident by the small standard variation in the mean pC:pN at these sites (Figure 3). The observed lower ratios can derive from a loss of food-derived carbon due to respiration, or by a proportionally greater content of phytodetrital nitrogen after feeding. A proportionally lower uptake of carbon compared to the other sites can be excluded as carbon uptake at 540/4 and 800/7 is not the lowest observed (Figure 2). Elevated pN-values could indicate growth of foraminifera, ingestion of phytodetritus-feeding bacteria, hosting of endobionts, or storage of phytodetrital nitrogen. Firstly nitrogen is an essential component of amino acids (proteins), and specific enzymes. The need of food-derived nitrogen for the synthesis of these compounds could be higher during foraminiferal growth which might have taken place during the experimental period in few specimens. Secondly, bacteria in the OMZ of the Indian Ocean also ingest *T. weissfloggii* (Hunter et al., 2012b) and their consumption by foraminifera (either selectively or together with phytodetritus) could have hence resulted in the presence and higher levels of pN in foraminifera, originating from bacteria. Simultaneous uptake of algae and bacteria was observed in *U*. ex. gr. *semiornata* in the OMZ of the Pakistan margin (Larkin et al., 2014). Equally hosting of denitrifying endobionts could also lead to higher pN levels in foraminifera, especially at 540 m where denitrification in bacteria is likely to occur. Endobionts capable of denitrification and storage of nitrate have been found in few species of foraminifera (Bernhard et al., 2012a,b) and specific bacteria can use organic nitrogen for denitrification under suboxic conditions (Gruber and Sarmiento, 1997). Because we did not investigate foraminifera for the presence of bacteria, we cannot exclude nitrogen uptake and its storage by endobionts. Thirdly, some species of foraminifera have been attributed to store nitrate and to perform denitrification (Risgaard-Petersen et al., 2006; Pina-Ochoa et al., 2010; Nomaki et al., 2015). Members of *Globobulimina, Bolivina*, and *Uvigerina* who showed nitrate storage in the deep Sagami Bay (Glud et al., 2009) showed high pN-values at 540 and 800 m (Table 2, Figure 4). Storage of organic nitrogen or its use for denitrification has not been shown for foraminifera so far but could explain the high pN-values under almost anoxic conditions at 540 m.

## Conclusions

In this study, the short-term response of calcareous foraminifera to a single pulse of diatom phytodetritus was studied at three different environments within the OMZ on the Indian Margin of the Arabian Sea. We showed that foraminifera assemblages reacted differently to food under different oxygen concentrations and that few species (e.g., *U. schwageri*) can dominate the carbon uptake of the assemblage. Different metabolic demands and feeding strategies as well as macrofauna presence seem to strongly effect the processing of organic matter by foraminifera in OMZ sediments. Highest uptake at the lowest oxygen concentrations and changes of uptake with water depth suggests that oxygen is not limiting foraminiferal uptake of phytodetritus but rather influencing total uptake by controlling distribution of the foraminiferal and macrofaunal species. The observed variation in food-derived carbon and nitrogen content in foraminifera shows different nutritional demands of foraminiferal species that are not visible when the experimental design focuses on only one nutrient (e.g., carbon). The use of double labeled algae can hence help to further identify physiological requirements in foraminifera. We were able to quantify organic nitrogen uptake of foraminifera at three depths in the OMZ of the Indian margin. Our results provide first insight in the cycling of nitrogen and possible storage of nitrogen from food but more data are needed to allow comparison and further conclusions. The marine nitrogen cycle is more scarcely investigated than the carbon cycle and especially so for the role of benthic foraminifera. Denitrification was only recently attributed to foraminifera in low oxygen environments (Risgaard-Petersen et al., 2006; Hogslund et al., 2008; Pina-Ochoa et al., 2010) and maybe extreme environments such as OMZs hold more specific metabolic adaptations for foraminifera.

## Author contributions

AE, acquisition of data, analysis, and interpretation of data, drafting manuscript; JW, acquisition of data, critical revision; UW, study conception and design; PH, acquisition of data.

### Conflict of interest statement

The authors declare that the research was conducted in the absence of any commercial or financial relationships that could be construed as a potential conflict of interest.
