# Supplementary material for: Carbon and Nitrogen Uptake of Calcareous Benthic Foraminifera along a Depth-Related Oxygen Gradient in the OMZ of the Arabian Sea
Source: Front Microbiol. 2016 Feb 11;7:71. doi: 10.3389/fmicb.2016.00071 (PMC4749719; doi:10.3389/fmicb.2016.00071)
Supplement: Supplementary file 1 [file Table1.pdf]

Supplementary table

Table S1. Total number of foraminiferal individuals analyzed in isotope analysis per sample, $\delta^{13}C$ & $\delta^{15}N$ (after incubation with labeled *T. weissflogii* for 4 to 10 days), and ratio of cytoplasmatic total organic carbon and nitrogen (TOC:TN) of living benthic foraminifera (> 125 µm) after feeding in the upper 1 cm sediment at 800 m and 1100 m.

Depth (m)	Duration (days)	Sample	# ind.	$\delta^{13}C$ (‰)	$\delta^{15}N$ (‰)	TOC:TN
800	4	Bolivinitidae	61	1414.4	1073.5	3.4
		Bulimina cf. *gibba*	55	61.9	311.9	6.1
		Bulimina cf. *gibba*	22	201.5	45.3	2.5
		Buliminidae (without *B.* cf. *gibba*)	71	378.3	466.4	3.7
		Cassidulinidae	64	88.9	355.2	3.1
		Epistominella rugosa	120	157.0	376.8	3.5
		Epistominella rugosa, *Epistominella exigua*	127	559.1	313.5	2.2
		Gyroidina spp., *Hoeglundina elegans*	35	7345.2	4833.4	3.6
		Pullenia quinqueloba	34	14.0	183.1	3.2
		Uvigerina schwageri, *Uvigerina bifurcata*	10	5846.7	4043.8	3.8
		remaining Uvigerinidae	90	10.0	234.5	5.1
		remaining calcareous	56	664.9	806.9	4.4
800	7	*Bolivina* aff. *B. dilatata*	19	2345.9	6549.9	2.3
		Bolivinitidae (without *B.* aff. *B. dilatata*)	68	137.3	876.2	3.7
		Bulimina cf. *gibba*	55	38.7	532.3	6.4
		Bulimina cf. *gibba*	43	498.8	3666.2	5.2
		Bulimina cf. *gibba*	38	63.8	213.2	3.6
		Buliminidae (without *B. gibba*)	61	286.6	1133.7	4.0
		Cassidulinidae	69	844.7	2718.0	3.2
		Chilostomella sp.	43	411.4	2317.5	4.3
		Epistominella rugosa	120	222.2	942.7	4.4
		Epistominella rugosa, *Epistominella exigua*	103	821.1	946.8	1.6
		Globobulimina sp.	49	101.2	551.3	3.0
		Gyroidina spp., *Hoeglundina elegans*	42	203.7	1327.4	4.1
		Lenticulina sp.	11	256.1	1639.5	3.2
		Pullenia spp.	29	517.6	3272.7	5.2
		Uvigerina schwageri, *Uvigerina bifurcata*	13	2942.7	8830.2	4.3
		Uvigerina semiornata	12	520.2	2554.2	2.7
		remaining Uvigerinidae	90	329.8	1544.2	4.5
		remaining calcareous	101	181.6	1203.1	5.0
800	10	Bolivinitidae	61	270.9	458.0	2.8
		Bulimina aculeata	31	371.9	686.5	1.7
		Bulimina cf. *gibba*	55	79.8	152.2	6.1
		Bulimina cf. *gibba*	64	36.8	135.0	4.9
		Buliminidae (without *B. aculeata*, *B.* cf. *gibba*)	89	44.2	165.1	2.8
		Cassidulinidae	104	25.7	178.5	3.9
		Epistominella rugosa, *Epistominella exigua*	120	790.6	741.1	3.8
		Epistominella rugosa, *Epistominella exigua*	98	291.1	219.2	2.2
		Epistominella rugosa	50	84.7	211.3	2.7
		Cancris sp., *Chilostomella* sp., *Globobulimina* sp.	19	42.4	356.9	4.7
		Gyroidina bradyi	8	529.2	632.6	1.7

		Hoeglundina elegans	9	62.7	281.5	2.5
		Lenticulina sp.	13	49.9	363.3	5.1
		Pullenia quinqueloba	22	83.5	296.5	3.0
		Uvigerina schwageri, Uvigerina bifurcata	25	6664.8	3806.6	3.6
		other Uvigerinidae	39	60.5	359.6	5.5
		other Uvigerinidae	125	176.3	426.3	5.0
		remaining calcareous	116	841.5	845.5	4.5
1100	4	*Chilostomella* sp., Buliminidae	84	207.9	220.5	3.2
		Globobulimina sp.	6	8.5	94.6	3.3
		spiral and one-chambered forms	64	247.0	278.0	3.3
1100	10	*Bulimina mexicana*	100	113.0	218.2	3.6
		Chilostomella sp., remaining Buliminidae	107	52.9	63.6	2.6
		spiral and one-chambered forms	35	10029.1	6086.2	3.9
